# Evaluation of podoplanin expression in vulvar squamous cell carcinoma and stromal fibroblasts and its prognostic implications

**DOI:** 10.1038/s41598-025-06908-8

**Published:** 2025-06-20

**Authors:** Gilbert Georg Klamminger, Meletios P. Nigdelis, Annick Bitterlich, Mariam Parvanta, Martin Ertz, Laura Schnöder, Bernd Holleczek, Roxana Schwab, Annette Hasenburg, Bashar Haj Hamoud, Erich Franz Solomayer, Mathias Wagner

**Affiliations:** 1 Department of General and Special Pathology, Saarland University (USAAR) and Saarland University Medical Center (UKS), Homburg, Germany; 2 Department of Obstetrics and Gynecology, University Medical Center of the Johannes Gutenberg University Mainz, Langenbeckstraße 1, 55131 Mainz, Germany; 3 Department of Gynecology, Obstetrics and Reproductive Medicine, Saarland University Medical Center (UKS), Homburg, Germany; 4 Saarland University Medical Center for Tumor Diseases (UTS), Homburg, Germany; 5 Saarland Cancer Registry, Saarbrücken, Germany

**Keywords:** Gynaecological cancer, Biomarkers

## Abstract

Although the immunohistochemical expression of the transmembrane sialoglycoprotein podoplanin (D2-40) has been repeatedly reported as a factor of prognosis in various tumor entities—its potential prognostic relevance in squamous cell carcinomas of the vulva remains to be evaluated. In the present study, we evaluated 68 patients with primary vulvar carcinomas (VC) and analyzed the cytoplasmic/membranous immunohistochemical expression of podoplanin in viable tumor cells and cancer-associated fibroblasts (CAFs) within the adjacent peritumoral stroma. To determine potential associations between podoplanin expression and traditional clinicopathological parameters (e.g., tumor stage, infiltration depth, groin lymph node metastasis), as well as with local recurrences, metastases, and patient’s overall survival, subsequent data analysis was conducted using Spearman correlation analysis, Fisher’s exact test and the log-rank test (Mantel-Cox). Although our results did not substantiate a positive correlation of podoplanin expression in tumor cells or CAFs with traditional histomorphological parameters, we propose a prognostic potential of podoplanin as a tissue-based biomarker in HPV-associated VC. In our exploratory pilot study, we observed a significantly improved overall survival in this distinct subgroup (Log-rank (Mantel-Cox) test; *p* = 0.0399). The results of our retrospective study may support the expectations that podoplanin, a common and widely available antibody, could be used as a tool for prognostic purposes in the future. Further research and validation are necessary before these markers can be implemented in clinical practice.

## Introduction

The academic-driven establishment of commonly available and easily applicable diagnostic and prognostic markers remains key in “orphan diseases” such as vulvar cancer (VC), where a variety of factors for instance the absence of particular screening programs as well as the challenge of enrolling a sufficient number of patients within clinical trials—just to name a few—have so far hindered effective improvements in terms of long-term survival^1^. Up to now, several approaches aimed to assess histomorphological aspects of the neoplasm, its (micro-)environment, or the interaction of them both in order to stratify the individual underlying biological tumor behavior. The prognostic relevance of the overall pattern of tumor infiltration, the morphological aspect of infiltrating nuclei, as well as distinct immunohistochemical aspects of an epithelial-to-mesenchymal transition (EMT) at the front of invasion were tested with partially promising results^2–5^.

Due to its common use as a diagnostic marker in routine surgical pathology for detection of lymphovascular space infiltration also in VC patients, the mucin-type sialoglycoprotein *podoplanin* (D2-40), is broadly available in almost all pathological laboratories and most research labs. Despite the specific labeling of the lymphatic endothelium, cancer cells as well as dendritic cells and especially cancer-associated fibroblasts (CAFs) express podoplanin to varying degrees—with poor clinical prognosis in case of high-rates CAFs within pulmonary adenocarcinoma as well as invasive breast cancers and a distinct relation with patient clinical outcome in case of tumoral D2-40 expression on cancer cells^6–11^. Despite the employment of the D2-40 antibody for the detection of intratumoral lymphatic vessel density in VC by Goes et al., a distinct evaluation of podoplanin expression in VC cells and corresponding CAFs has—to our knowledge—not yet been established^12^. In the present study, we systematically examined the clinical relevance and the prognostic role of both podoplanin-expressing tumor cells as well as podoplanin expressing CAFs in HPV-associated and HPV-independent squamous cell carcinomas of the vulva.

## Material and methods

### Study cohort

In this study, 68 patients who underwent vulvectomy for squamous cell carcinoma of the vulva were included. Each patient subsequently received a detailed diagnostic pathological evaluation of the surgical specimen at the University Hospital of Saarland in Homburg, Germany. Patients were identified in cooperation with the Saarland University Medical Center for Tumor Diseases (UTS) registry by searching the hospital’s internal databases using the ICD-O codes 8085/3, 8086/3, and 8070/3. As a priori defined, we excluded patients with solely high-grade squamous cell intraepithelial neoplasias (HSIL) and neoplasms of the vulva other than squamous cell carcinomas (e.g., adenocarcinomas, melanomas). Additionally, recurrent cancerous affections as well as patients who underwent only palliative surgery were excluded. Available data on survival, occurring local recurrences, and metastases were retrieved by the statewide-operating cancer registry ‘Saarland Cancer Registry’. The study was approved by the Ethics Committee of Saarland (study identification number 249/23, approved on 07. March 2024) and was performed according to the Declaration of Helsinki^13^.

### Immunohistochemical stainings and interpretation

Immunohistochemical analysis was employed to assess the expression patterns of podoplanin in epithelial neoplastic cells and cancer-associated fibroblasts (CAFs). Therefore, the surgical specimens were formalin fixed, paraffin embedded and cut into three 4 µm thin cuts (Leica RM 2235 rotation microtome, Leica Microsystems, Wetzlar, Germany), which were mounted on slides (Matsunami TOMO) using a water bath (46 °C), and consecutively stored at 37 °C. Subsequent H&E staining was performed and served as a morphological control. For the immunohistochemical analysis, a primary antibody specific for podoplanin (mouse monoclonal antibody, Dako/Agilent, Santa Clara, CA, USA; clone D2-40) was applied for 32 min at 37 °C using the Benchmark Ultra (Ventana Medical Systems). Antibody binding was detected employing the ultraView Universal Alkaline Phosphatase Red Detection (Roche, Basel, Switzerland) in alignment with the manufacturer’s instructions and antigen retrieval at 97 °C (heat-induced) was performed using CC1 buffer (Ventana) for 64 min. Every staining series incorporated negative controls by waiving the primary antibody; lymphatic endothelium served as the internal on-slide positive control.

Furthermore, available p16 stainings of the associated diagnostic cases were re-evaluated as part of the study (n = 60); as defined by the ‘WHO Classification of Female Genital Tumors’, it hereby served as a surrogate marker for HPV association^14^, and a ‘block type’ p16 expression of at least 20 neighboring p16-expressing tumor cells was considered as HPV positive (HPV-associated VC). Tumors with a ‘patchy’ (irregular, nuclear and cytoplasmatic stainings distributed in a heterologous manner) p16 expression or the absence of its immunohistochemical expression were considered as HPV-independent.

**Fig. 1:**
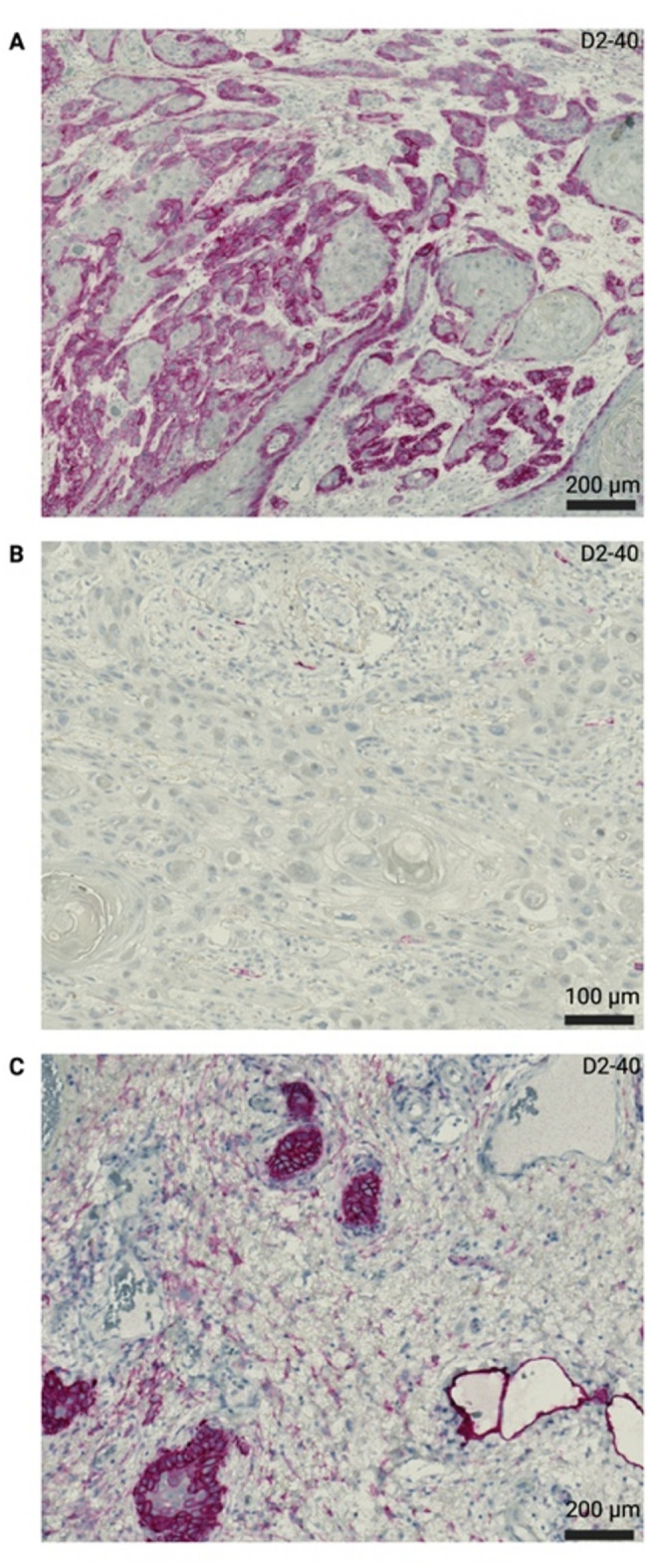
Display of immunohistochemical podoplanin expression types. (**A**) ‘Podoplanin positive’ expression type with > 2% viable tumor cells showing a cytoplasmatic staining pattern. (**B**) ‘Podoplanin negative’ expression type with ≤ 2% viable tumor cells showing cytoplasmatic staining pattern. (**C**) Within the peritumoral stroma > 10% of the fibromatous component showed podoplanin-expression (‘CAFs podoplanin positive’). As a regular on-slide positive control, podoplanin positive lymphatic endothelium can be seen in the right half of the image.

As a first step in the analysis, the overall percentage of cytoplasmatically/membranously podoplanin stained tumor cells was determined; to further evaluate a potential prognostic impact of podoplanin, a ‘podoplanin positive’ cohort with > 2% protein expression and ‘podoplanin negative’ cohort with ≤ 2% protein expression in viable tumor cells were defined. According to the analysis of CAFs by the team of Schoppmann et al., the CAFs at the invasive tumor front were rated as either ‘CAFs podoplanin positive’ in case > 10% of the fibromatous component of the peritumoral stroma expressed podoplanin or ‘CAFs podoplanin negative’ in case the podoplanin expression of the fibromatous component of the peritumoral stroma was less than 10%. Figure 1 outlines concrete examples of our defined cohorts. In order to reduce inter-observer variability, histomorphological analyses were performed collaboratively using a multi-head light microscope (GGK, AB, MP, MN), and upcoming discrepancies were addressed through consensus-based discussions (MW).

### Statistical analysis

All data were stored in an Excel file and imported into GraphPad (Boston, MA 02110, US) for calculations. Spearman correlation analysis was used to assess the statistical relationships between percentage of podoplanin expression and clinicopathological variables. Positive correlations were tested against the null hypothesis of being due to random sampling. In a next step Fisher’s exact test was employed to test for differences in recurrence and metastasis between our priori defined groups and consecutively Log-rank (Mantel-Cox) test was used to test for the prognostic impact of podoplanin expression in CAFs and tumor cells on overall survival. Solely results with α < 0.05 were considered as statistically significant.

## Results

### Patient data

Our cohort comprises a total of 68 patients with primary surgically treated VC (median age 65.5 years [52–78.5 years, 25%-75% Percentile]). Among these, 11 patients presented with tumor stage T1a, 44 with T1b, 12 with T2, and 1 with T3. Overall, 16 patients showed involvement of the inguinal lymph nodes (videlicet at least N1 according to the actual TNM guidelines, 8th edition 2018), while 52 patients had no affection of the groin lymph nodes. During an average follow-up period of 60.1 months, 14 patients developed a locoregional recurrence, 6 patients experienced distant metastasis, and 22 patients died. Refer to Table 1 for a summary of the clinical information of our study cohort.

**Table 1:** Overview of clinical baseline characteristics within our study group. CI: confidence interval.

Clinical parameter of interest	n = 68 (percentage)
T1a	11 (16%)
T1b	44 (65%)
T2	12 (18%)
T3	1 (1%)
N0	52 (76%)
Positive groin lymph nodes (N1a/1b/2a/2b/2c)	16 (24%)
Infiltration depth	0.74 cm (mean; CI: 0.54–0.94 cm)
Lymphovascular infiltration (L1)	10 (15%)
Perineural infiltration (Pn1)	8 (12%)
Vascular infiltration (V1)	5 (7%)
HPV-association VC	18 (26%)
HPV-independent VC	42 (62%)
VC NOS (not otherwise specified)	8 (12%)
Local recurrence	14 (20%)
Metastasis	6 (9%)

### Correlation analysis of D2-40 expression with classic pathomorphological parameters

Our preliminary correlation analysis did not identify any significant correlations of the amount of D2-40 expression/CAFs and considered clinicopathological parameters; hereby, they were neither associated with tumor stage, infiltration depth, amount of groin lymph node affection, nor histological tumor grade, see Tables 2 and 3 for Spearman r and two-tailed *p*-values of each individual analysis. Additionally, the amount of podoplanin-expressing tumor cells was not associated with D2-40 positive CAFs (r = 0.1635 [CI: − 0.08511 to 0.3929]; *p* = 0.1828).

**Table 2:** Spearman correlation analysis of the amount of podoplanin expression on viable tumor cells with selected histological parameters.

Spearman correlation: Podoplanin expression on tumor cells in association to -	tumor stage	infiltration depth	amount of groin lymph node affection	histological tumor grade (grade I–IV)
r	-0.1012	-0.1053	-0.01804	0.02053
95% confidence interval	-0.3380 to 0.1476	-0.3416 to 0.1436	-0.2657 to 0.2319	-0.2258 to 0.2644
*p* value (two-tailed)	0.4114	0.3930	0.8857	0.8680

**Table 3:** Spearman correlation analysis of the expression of podoplanin on CAFs with selected histological parameters.

Spearman correlation: Podoplanin expression on stromal fibroblasts in association to -	tumor stage	infiltration depth	amount of groin lymph node affection	histological tumor grade (grade I–IV)
r	0.02707	-0.06024	0.1062	0.1863
95% confidence interval	-0.2196 to 0.2705	-0.3010 to 0.1877	-0.1466 to 0.3459	-0.06172 to 0.4126
*p* value (two-tailed)	0.8266	0.6256	0.3962	0.1282

### Impact of D2-40 expression on recurrence, metastasis, and survival

**Fig. 2:**
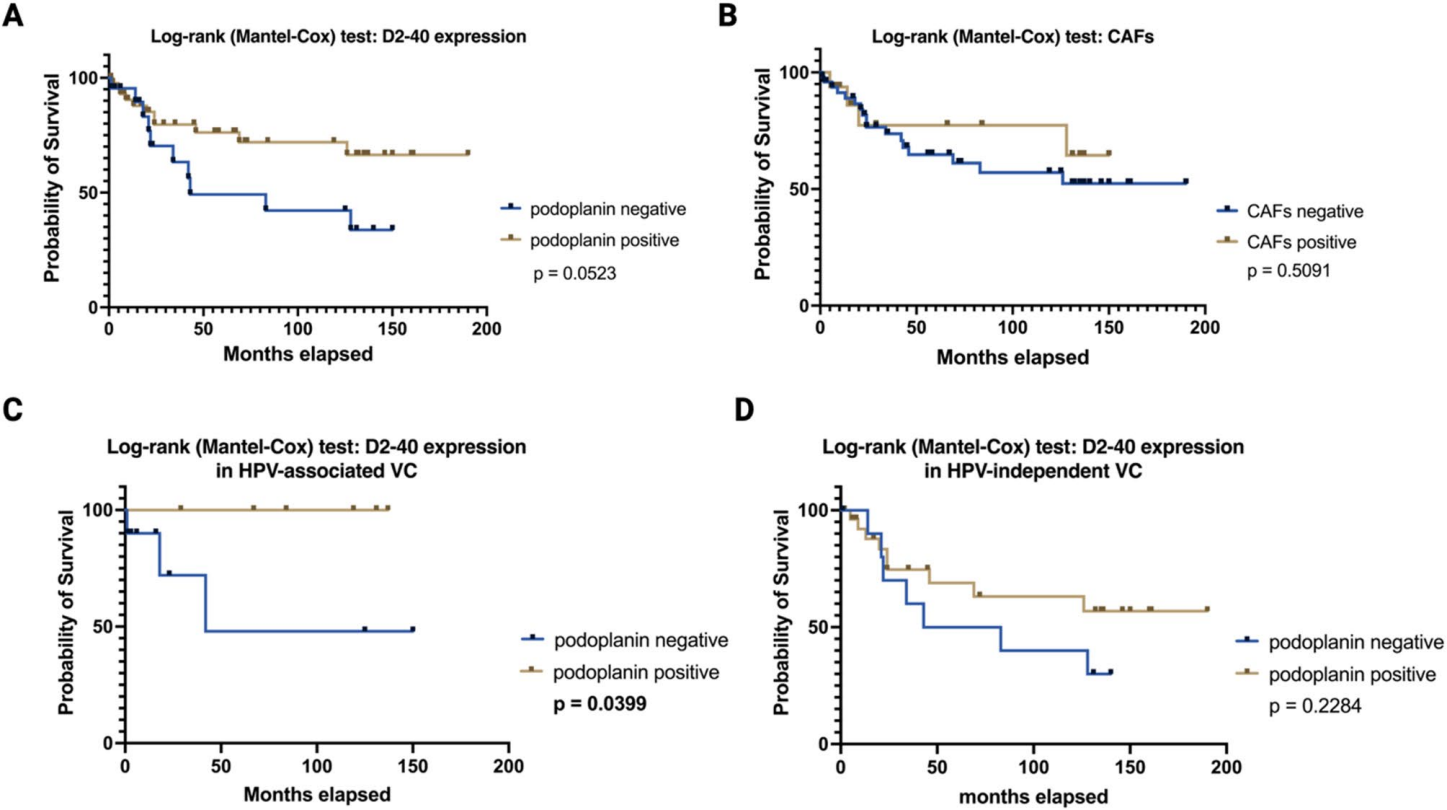
Survival of VC patients stratified by podoplanin-expression in cancer cells and CAFs. Although within our study cohort overall survival was initially neither related to podoplanin (podoplanin positive vs. podoplanin negative tumors) expression (**A**) nor the amount of podoplanin positive CAFs (**B**), a subsequent subgroup analysis determined the prognostic potential of podoplanin positive HPV-associated tumors (Log-rank (Mantel-Cox) test; ***p* = 0.0399**, x^2^ = 4.223; (**C**). Interestingly, such an effect could not be observed in patients with HPV-independent tumorigenesis (**D**).

Within our cohort, neither recurrence risk nor the development of metastasis differed significantly with regard to podoplanin expression (Fisher’s exact test; *p* =  ≥ 0.9999) or amount of podoplanin positive CAFs (Fisher’s exact test; *p* = 0.2909 and *p* = 0.6201 respectively). Furthermore, overall survival was not distinctively impaired in relation to the immunohistochemical expression of podoplanin within neoplastic cells (Log-rank (Mantel-Cox) test; *p* = 0.0523, x^2^ = 3.767) or podoplanin-positive CAFs (Log-rank (Mantel-Cox) test; *p* = 0.5091, x^2^ = 0.4359); see Fig. 2A, B. Nevertheless, consecutive further subgroup analyses—achieved by splitting the study cohort with regard to inguinal nodal involvement (N0 vs. N-positive), tumor stage (locally confined tumors defined as T1a and T1b vs. local anatomical structures infiltrating tumors defined as T2 and T3), and HPV status (positive vs. negative)—suggested a significant impact on survival in the cohort of HPV-associated VC. Here, patients with an expression of podoplanin in viable tumor cells (‘*podoplanin positive’*) showed a significantly better prognosis (Log-rank (Mantel-Cox) test; ***p* = 0.0399**, x^2^ = 4.223); the analysis of CAFs did not play a prognostic role in any of the subgroups put to test. Table 4 and Fig. 2C, D visualize the association of podoplanin expression with regard to selected (sub-)groups.

**Table 4:** Depicts the survival analysis of podoplanin positive vs. podoplanin negative VC with regard to different subgroupings.

Survival analysis: Log-rank (Mantel-Cox) test	*p*-value	x^2^ value
D2-40 expression (pos/neg) within N0 tumors	*p* = 0.0803	x^2^ = 3.059
D2-40 expression (pos/neg) within lymph node positive tumors	*p* = 0.3869	x^2^ = 0.7488
D2-40 expression (pos/neg) within locally confined tumors (T1a and T1b)	*p* = 0.1830	x^2^ = 1.733
D2-40 expression (pos/neg) within local anatomical structures infiltrating tumors (T2 and T3)	*p* = 0.2208	x^2^ = 1.499
D2-40 expression (pos/neg) within HPV-associated tumors	***p* = 0.0399**	x^2^ = 4.223
D2-40 expression (pos/neg) within HPV-independent tumors	*p* = 0.2284	x^2^ = 1.451

## Discussion

In the present study, we systematically examined the immunohistochemical expression of podoplanin as an easily applicable and broadly available biomarker in not only viable vulvar carcinoma cells but also peritumoral fibroblasts. Hereby, we did not only consider biologically and histologically established risk factors such as the presence of inguinal lymph node metastases or the depth of infiltration but also we evaluated the prognostic relevance of D2-40 expression with regard to the underlying tumor entity and its respective (viral) tumorigenesis, namely in HPV-associated VC and HPV-independent VC.

Comparable approaches in diverging tumor entities have already been described in the literature—albeit with so far inconclusive evidence^9,15–19^. With respect to squamous cell carcinomas of the oral cavity a recent review by Mello et al. identified 22 cohort and seven cross-sectional studies that analyzed the prognostic impact of D2-40 expression within tumor cells on patient`s survival and/or clinicopathological variables^15^. Four out of twelve studies determined a general negative association between podoplanin expression and overall survival^9,16,17,20^. Hu et al. systematically reviewed the prognostic value of D2-40 within lung squamous cell cancer and included 8 studies in a comprehensive metanalysis. Although they determined a high correlation of its immunohistochemical expression with tumor differentiation, they did not identify any correlation with lymphatic and vascular invasion or metastasis; interestingly, they postulate a positive (better) association between podoplanin expression and survival^21^. Within squamous cell carcinoma of the skin podoplanin expression was reported to be associated with higher risk of lymph node metastasis and lymphovascular invasion^22^ and shorter survival^18,23^. Interestingly, Neinaa et al. showed a varying D2-40 expression depending on the underlying tumor entity (basal cell carcinoma, squamous cell carcinoma, basosquamous carcinoma); nevertheless, all examined tumor types differed in their higher podoplanin expression in contrast to physiological control epidermis. From a pathophysiological point of view, podoplanin as a transmembrane glycoprotein may be linked in particular to processes of EMT, cell migration, and platelet aggregation via interactions with the cell–cell adhesion protein E-cadherin and the C-type lectin-like receptor 2^24–27^.

In our study, we neither detected any impact of podoplanin expression on classical prognostic parameters such as infiltration depth, nor did we find a clear association with the risk of local recurrence or metastasis in VC. Furthermore, we did not find a relevant use case for podoplanin staining of CAFs in VC—in contrast to preliminary findings, for example in breast cancer^8^. Even though the expression of D2-40 was not related to overall survival in the initial log-rank analysis within our cohort, a consecutive subgroup analysis proposed a significant prognostic potential within HPV-associated tumors, where patients with ‘podoplanin positive’ tumors had a favorable prognosis in comparison to ‘podoplanin negative’ tumors. In terms of their clinical utility, the results facilitate the identification of a subgroup with arguably good prognosis, which could be quickly and routinely inferred from a block-type p16 positivity (HPV association) and existing podoplanin expression.

Since there is great disagreement in the existing literature regarding the optimal cut-off of a defined podoplanin expression, we decided against an arbitrary value, which would divide interpretations/quantifications of the stainings into different groups of podoplanin expression (high/medium/low). In comparison, we opted for a cut-off that follows the all-or-nothing principle (either negative or positive). We attached particular importance to not directly define the value ‘0%’ as a negative cut-off since our selected cut-off of ≤ 2% as ‘negative’ allowed not only further reduction of potentially occurring interobserver variabilities but also avoids confusion with regular staining artifacts. In our opinion, alternative binary cut-off values, of for example, 5%, 10%, 20%, and 50%, do not sufficiently reflect this consideration and were therefore not adopted for our analysis^16,17,28,29^. Nevertheless, it is important to note that conflicting results of the prognostic ability of podoplanin expression were repeatedly reported^15,21^ and it remains to be resolved if differing results and conclusions arise from either laboratory, diagnostic or biological variations in the different studies. When interpreting our results, several potential limitations must be considered, including the sample size, the retrospective study design, and the exploratory nature of the study. Further external validation is required before these findings can be translated into clinical practice. Furthermore, future studies could explore the relationship between distinct patterns of tumor infiltration (e.g., pushing vs. spray-like invasion) and podoplanin expression. Looking ahead, only a uniformity of the methodology and the creation of an experimental-methodological consensus regarding e.g., specific antibodies and interpretations of the expression analysis would allow for inter-institutional comparability and subsequently can guarantee a higher level of evidence in a next step.

In conclusion, our pilot study suggests the prognostic value of the widely available immunohistochemical marker podoplanin for assessing the prognosis of HPV-associated vulvar epithelial neoplasia. However, further studies are needed to validate the results and further compare the expression of podoplanin in VC also with additional clinicopathological parameters such as PD-L1 expression or genomic alterations. Nevertheless, the search for new potential tissue-based biomarkers and individualized treatment approach in HPV-associated VC is particularly reasonable for younger patients, who are often affected by a virus-dependent pathogenesis.

## Data Availability

Interested parties are warmly invited to contact the corresponding author for individual options.
